# Fibrome utérin associé à un STUMP (tumeurs musculaires lisses à potentiel de malignité incertaine): à propos d'un cas

**DOI:** 10.11604/pamj.2018.31.151.14135

**Published:** 2018-10-30

**Authors:** Mohammed Karam Saoud, Imane Benchiba, Nisrine Mamouni, Sanaa Errarhay, Chahrazad Bouchikhi, Abdelaziz Banani

**Affiliations:** 1 Service Gynécologie-Obstétrique I, CHU Hassan II, Fès, Maroc

**Keywords:** Tumeurs musculaires lisses à potentiel de malignité incertain (STUMP), léiomyome, léiomyosarcome, Smooth muscle tumor of uncertain malignant potential (STUMP), leiomyoma, leiomyosarcoma

## Abstract

Les tumeurs musculaires lisses à potentiel de malignité incertain (STUMP) sont des tumeurs musculaires lisses dont les caractéristiques morphologiques ne permettent pas de les ranger de façon formelle en tumeur bénigne ou maligne. Nous rapportons ici le cas d'une patiente âgée de 44 ans sans antécédents pathologiques notables qui consulte pour une augmentation du volume abdominal. Une échographie pelvienne et une TDM TAP ont été réalisées objectivant une énorme masse à double composante tissulaire et liquidienne au dépend de l'utérus. La patiente a bénéficié d'une laparotomie exploratrice avec découverte de deux masses, une au dépend de l'utérus et l'autre en rétropéritonéal. Le résultat anatomo-pathologique était en faveur d'un myome utérin associé à un STUMP.

## Abstract (en)

STUMP (smooth muscle tumor of uncertain malignant potential) are smooth muscle tumors that cannot be diagnosed as either benign or malignant due to their morphological characteristics. We here report the case of a 44 year old patient with no notable medical history presenting with an increase in the abdominal volume. Pelvic ultrasound and CT TAP scan were performed showing voluminous mass with tissue and fluid-filled component in the uterus. The patient underwent exploratory laparotomy during which two masses were detected, one in the uterus and the other in the retroperitoneal area. Anatomopathologic examination showed uterine myoma associated with a STUMP.

## Introduction

Les tumeurs musculaires lisses de l'utérus sont fréquentes et majoritairement bénignes. Certaines tumeurs peuvent présenter des aspects anatomo-pathologiques inhabituels entrainant des problèmes de diagnostic différentiel notamment avec le leiomyosarcome. Ainsi, L'OMS a classé ces tumeurs en 2003 en tumeurs musculaires lisses à potentiel de malignité incertain (STUMP).

## Patient et observation

**Figure 1:**
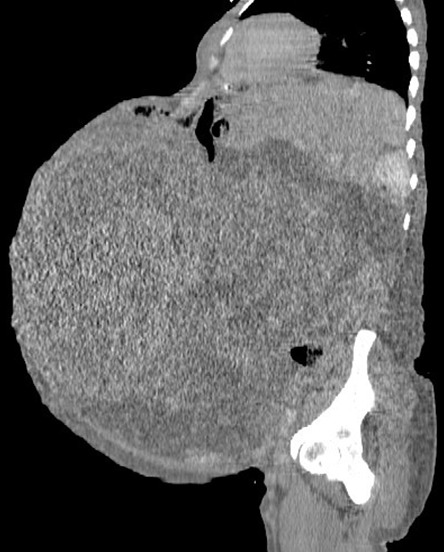
Coupe sagittale d’une TDM TAP C- montrant une énorme masse abdomino-pelvienne à triple composante calcique, kystique et solide refoulant tout le digestif en haut. Utérus et ovaires non vus

**Figure 2:**
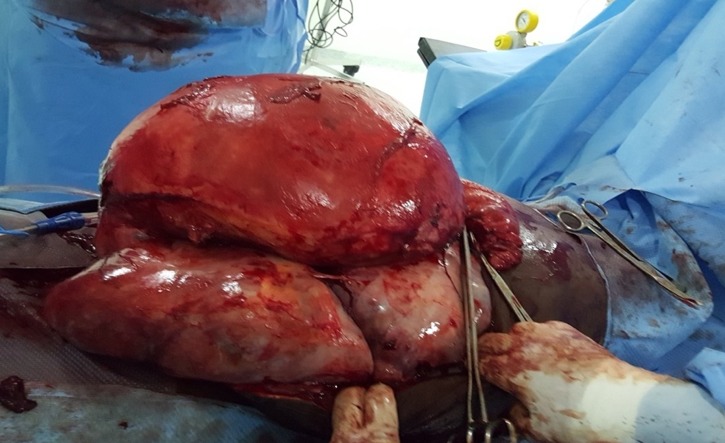
Photo per opératoire de 2 masses superposées, la première au dépend de l’utérus et la deuxième en rétro péritonéale

**Figure 3:**
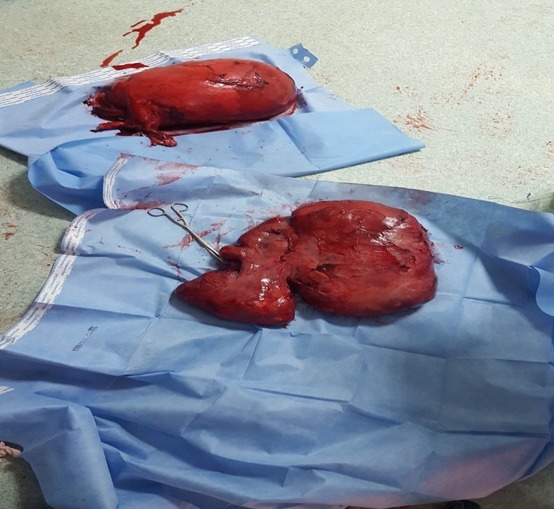
Photo des 2 masses après leurs ablations

Il s'agit d'une patiente âgée de 44 ans, sans antécédents pathologiques notables, célibataire et nulligeste, toujours réglée avec des cycles réguliers. Référée dans notre formation pour prise en charge d'une augmentation du volume abdominal associée à des douleurs abdomino-pelviennes évoluant depuis 8 mois sans autres signes associés notamment des signes de compression ou envahissement urinaire et digestive. L'examen clinique à l'admission trouve une patiente consciente, normo tendu, normo carde, eupnéique et apyrétique. L'examen abdominal trouve une volumineuse masse dure arrivant jusqu'à l'appendice xiphoïde. L'examen gynécologique (spéculum + toucher vaginal) non fait car la patiente se dit vierge. Au TR énorme masse abdomino-pelvienne mobile difficilement caractérisable. La patiente a bénéficié d'une échographie pelvienne dans notre formation objectivant une énorme masse tissulaire avec des zones kystiques prenant le doppler couleur et occupant la totalité de l'écran: utérus et les deux ovaires non vus; présence d'une faible lame d'ascite. Une TDM TAP a été réalisée (Figure 1), elle montre une énorme masse abdomino-pelvienne à triple composante calcique, kystique et solide, ovaires et utérus non vus, présence d'ascite de grande abondance et absence de localisation secondaire. La patiente a bénéficié d'un bilan pré opératoire et d'une visite pré anesthésique et fut programmée pour une laparotomie exploratrice. L’incision utilisée était xipho pubienne vu la taille de la masse, à l'exploration (Figure 2, Figure 3): ascite de moyenne abondance, utérus augmenté de taille siège d'une masse fundique de 40cm à paroi blanchâtre lisse et rénitente, reposant sur une énorme masse kystique de 50cm arrivant jusqu'à l'appendice xiphoïde à paroi lisse sans végétation ni granulation exo kystique situé en rétro péritonéale. Les deux annexes et le reste de la cavité abdomino-pelvienne sont sans particularité.

**Figure 4:**
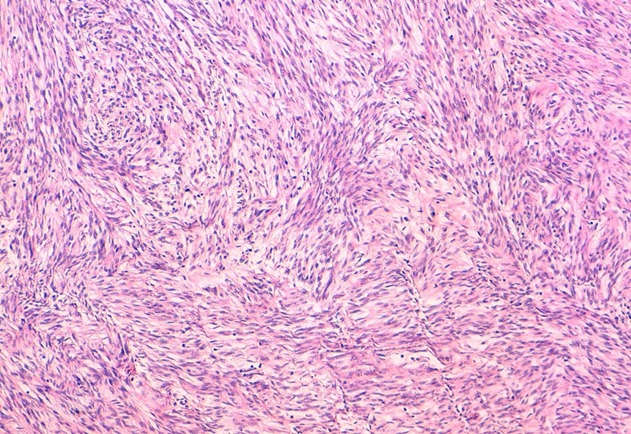
Prolifération des de faisceaux musculaires lisses entrecroisés à angle droit (HESx100)

**Figure 5:**
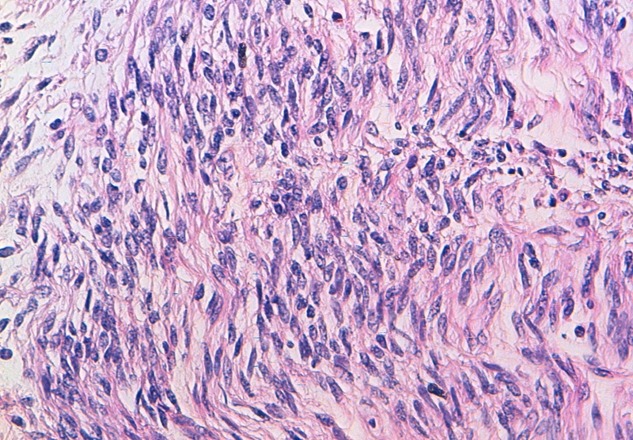
Prolifération des cellules allongées avec quelques atypies et mitoses (HESx400)

La patiente a bénéficié dans un premier temps d'une hystérectomie totale + annexectomie bilatérale qui était difficile vu les multiples adhérences utéro-colique et utéro-grelique. Puis les chirurgiens viscéralistes ont terminé le geste en disséquant laborieusement la masse rétro-utérine en respectant le tube digestif dont elle présentait avec des adhérences intimes. L'acte opératoire a duré 5h et la patiente a nécessité une transfusion de 3CG O+ et 4PFC. Le résultat anatomo-pathologique des deux pièces était le suivant: la 1^ère^ pièce au dépend de l'utérus: léiomyome en nécrobiose aseptique et la 2^ème^ pièce rétro-péritonéale (Figure 4, Figure 5): il s'agit d'une prolifération tumorale d'architecture fasciculée. Elle est faite de faisceaux musculaires lisses entrecroisés à angle droit et séparés par des trousseaux de collagène. Les cellules tumorales sont allongées, présentant des atypies significatives focales avec un index mitotique estimé à 7 mitoses/10 champs au fort grossissement (CFG), présence de foyers de nécrose. La tumeur correspond à une tumeur musculaire lisse à potentiel de malignité incertain (STUMP). Le cas fut staffé en réunion de concertation pluridisciplinaire avec les médecins oncologues, radiothérapeutes et radiologues, la décision était une surveillance clinique et échographique chaque 3 mois. La patiente a bénéficié de 4 contrôles cliniques et échographies sans particularité avec une TDM TAP qui n'a pas objectivé des localisations secondaires.

## Discussion

Sur le plan morphologique, il existe un éventail de tumeurs musculaires lisses utérines comptant des issues cliniques prévisibles et des critères histologiques conventionnellement bien définis; aux deux extrémités de cet éventail se trouvent les léiomyomes et les léiomyosarcomes. Entre ces deux extrémités se trouvent plusieurs variantes qui comptent des caractéristiques histologiques, une évolution clinique et un pronostic inhabituel. L'Organisation Mondiale de la Santé les a qualifiées de tumeurs musculaires lisses à potentiel de malignité incertain [1]. Les STUMP sont des tumeurs très rares, il n'existe malheureusement pas des pourcentages exacts concernant leurs incidences de survenue, dû certainement au faible nombre des différentes séries. Sur le plan histologique [2], trois critères permettent de classer les tumeurs musculaires lisses en tumeurs bénignes ou malignes. Ces critères comprennent les atypies nucléaires, l'index de mitose et la présence de nécrose tumorale. Ainsi les leiomyosarcomes sont définis par la présence de cellules fusiformes avec: atypies nucléaires modérées à sévères, plus de dix mitoses sur dix champs au grossissement 40 X, présence d'une nécrose tumorale. Deux de ces critères sont nécessaires pour retenir le diagnostic de malignité. On parle de STUMP lorsqu'un des critères de malignité est présent et le second est difficile à évaluer. Ainsi on regroupe les cas suivants dans la catégorie des STUMP: une tumeur musculaire lisse à cellules fusiformes avec des atypies nucléaires modérées à sévères et un index mitotique limite entre huit et neuf mitoses, une tumeur musculaire lisse à cellules fusiformes avec des atypies nucléaires modérées à sévères et une nécrose dont la nature tumorale ou ischémique est difficile à évaluer, une tumeur musculaire lisse à cellules fusiformes avec plus de dix mitoses et une nécrose dont la nature tumorale ou ischémique est difficile à évaluer une vraie nécrose tumorale dans un léiomyome banal.

Dans notre cas, l'étude anatomo-pathologique a objectivé un index mitotique estimé à 7 mitoses/10 champs au fort grossissement (CFG) avec présence de foyers de nécrose la classant dans la catégorie des STUMP. Suspecter le diagnostic cliniquement ou radiologiquement reste difficile vu que les STUMP ne présentent pas de caractères spécifiques comparés au léiomyome bénin. Néanmoins pour la recherche de métastases, l'étage thoraco-abdomino-pelvien doit être systématique vu que des cas de métastases notamment pulmonaire suite à des STUMP ont été rapportés dans la littérature [3, 4]. En revanche, il n'y a pas de données précises sur le caractère métastatique des STUMP. Dans notre cas, jusqu'à présent une seule TDM TAP a été réalisée n'objectivant aucune localisation secondaire [3]. Cette incertitude dans le diagnostic anatomo-pathologique mène fréquemment à des dilemmes thérapeutiques, particulièrement lorsque le diagnostic est établi à partir de prélèvements de myomectomie issus des femmes qui souhaitent maintenir et/ou améliorer leur fertilité. Ainsi la conduite thérapeutique diffère entre les différentes écoles, entre simple myomectomie à une hystérectomie totale voir même associée à une annexectomie bilatérale. Le cas de notre patiente, la myomectomie était difficilement réalisable vu le caractère saignant du geste et les multiples adhérences que présentait la masse avec le digestif ainsi que son importante taille. Le pronostic des STUMP est meilleur que celui des leiomyosarcomes. Les différentes études (Tableau 1) ont constaté un taux de récidive nettement diminué par rapport au leiomyosarcome ainsi qu'une survie à 5 ans allant de 92 à 100% des patientes [2]. Par ailleurs des cas de métastases notamment pulmonaire en étaient rapportés, on rapporte probablement avec la propagation hématologique préférentielle des leiomyosarcomes [5]. Vu le caractère et l'évolution incertains de ces tumeurs, les patientes devraient recevoir un suivi rapproché et de longue durée clinique et radiologique [6, 7]. Notre patiente a bénéficié d'un examen clinique et d'une échographie pelvienne chaque 3 mois et d'une TDM TAP chaque an.

**Tableau 1:** Suivi et récidives des STUMP dans les différentes études

Séries	Nombre de patiente	Traitement	Suivi par mois	Récidive
Anderson et al. [4]	41	Hystérectomie totale ou myomectomie	16 et 63 mois	3 récidives
				STUMP pelvien
				métastase pulmonaire
				leiomyosarcome
Andrea et al. [6]	8	Hystérectomie totale ou hystérectomie totale +	22 et 123 mois	1 récidive: métastase pulmonaire
Berretta [7] et al.	1	Annexectomie bilatérale ou myomectomie	108 mois	Pas de récidive
Notre cas	1	Hystérectomie totale + annexectomie bilatérale + ablation de la masse rétropéritonéale	19 mois et toujours suivie	Pas de récidive

## Conclusion

Les STUMP correspondent à une entité histopathologique bien particulière. Leur diagnostic anatomo-pathologique est délicat, nécessitant un long suivi clinique et radiologique afin de confirmer la nature bénigne ou maligne de la tumeur. Leur prise en charge thérapeutique pose un problème notamment quand la femme est désireuse de préserver sa fertilité. Le pronostic des STUMP reste intermédiaire entre celui des léiomyosarcome et léiomyome.

## Conflits d’intérêts

Les auteurs ne déclarent aucun conflit d’intérêts.
